# Estimation of Parental Abundance Using Hierarchical Bayesian Modeling With Data Augmentation

**DOI:** 10.1002/ece3.73131

**Published:** 2026-03-12

**Authors:** Benjamin Marcy‐Quay, Nicholas M. Sard

**Affiliations:** ^1^ U.S. Geological Survey, Great Lakes Science Center Hammond Bay Biological Station Millersburg Michigan USA; ^2^ Biological Sciences Department SUNY Oswego Oswego New York USA

**Keywords:** abundance estimation, kinship, pedigree accumulation, population dynamics, rarefaction, semelparity

## Abstract

Pedigree‐based estimation methods leverage the fact that each offspring in a cohort is genotypically “marked” by its parents and represent a recent and promising toolset for estimating population dynamics. This includes pedigree accumulation estimators that model the “accumulation” of inferred unique parents within a given cohort to estimate parental abundance. Unlike close‐kin mark‐recapture approaches, which rely on intercohort comparisons, pedigree accumulation modeling can be completed solely using intracohort samples. This is particularly advantageous for semelparous species, where intercohort pairs are impossible and adult life stages can be difficult to sample without affecting their likelihood of successfully reproducing. Previous work has evaluated a range of estimators for such datasets, concluding that the non‐parametric Chao estimator provides the most accurate and precise estimates for feasible levels of sampling effort. We used simulated data to evaluate an alternative estimator based on hierarchical modeling and data augmentation in a Bayesian framework. Results indicate that estimates from the hierarchical Bayesian estimator had comparable accuracy and better precision than both the previously tested Chao1 estimator and the improved iChao formulation across a range of sample sizes and sex ratios. Furthermore, the Bayesian estimator was far more robust to simulated errors in pedigree reconstruction, especially the presence of false negatives. Hierarchical Bayesian pedigree accumulation models can also provide additional insight into underlying reproductive ecology through their use of an explicit observation process, allowing for the incorporation or estimation of species‐ and population‐specific reproductive dynamics. More broadly, the parametric nature of these models offers opportunities to efficiently pool information among datasets as well as to propagate uncertainty within more complex models.

## Graphical abstract

Pedigree accumulation estimators leverage the fact that each offspring in a cohort is genotypically “marked” by its parents and represent a recent and promising toolset for estimating population dynamics. We developed a hierarchical Bayesian pedigree accumulation estimator and compared it to previously evaluated non‐parametric approaches. Results indicate that the new method is similarly accurate, more precise, and capable of providing additional ecological insights versus previous approaches.

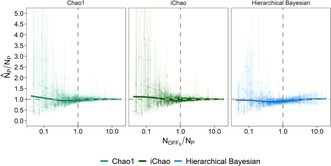

## 1 Introduction

Although considerable debate and uncertainty exist regarding the formulation and impact of stock‐recruit relationships (Subbey et al. 2014), estimates of spawning stock abundance have long been recognized as crucial to conservation and management (Beverton and Holt 1957; Ricker 1954). Estimating spawning stock abundance is particularly important for semelparous species, with the extreme represented by Pacific salmon. Consider that winter‐run Chinook salmon ( *Oncorhynchus tshawytscha* ) exhibit such high natal fidelity that they only exist in a single river (Waples et al. 2004). However, estimates of spawning stock are also especially tricky for such species as spawning leads to an individual's death, rendering it unobservable. From a practical perspective, individuals are most likely to be captured before or after spawning. Sampling before can disrupt spawning and leave their eventual success unknown, while sampling after spawning is often infeasible due to challenges associated with rapid decomposition and scavenging.

Pedigree‐based estimation methods have recently emerged as a promising solution for studying this difficult‐to‐observe life stage. In effect, spawner stock abundance can also be considered the number of parents that produced a set of offspring during a breeding season, which is hereafter referred to as *N*_P_, after Waples (2025). We use *N*_P_ rather than *N*_s_ (the number of successfully reproducing adults, with “S” representing success), as described in Sard et al. (2021), because *N*_P_ is simply a clearer and more efficient descriptor of the parameter. The approach leverages the fact that each offspring in a cohort is genotypically “marked” by its parents; although the parents themselves are not observed, an offspring represents the product of exactly two successfully spawning individuals. By inferring full‐ and half‐sibling pairs within a sample of offspring, this can be further extended to estimate the minimum number of successful spawners represented by the sample as well as the distribution of offspring among those parents. These data resemble those collected during surveys of species richness, where multiple observations of a site yield sightings (and resightings) of occupying species, and the challenge is to estimate the total number of species, including those “rare” species that are never sighted. Accordingly, techniques from this literature have been adapted to estimate abundance relying on the “accumulation” of unique genotypes as the sample size rises (or, conversely, the increasing “rarefaction” of unobserved genotypes in the unsampled pool). Initially, this was used on individual (rather than parental) genotypes sampled through fecal DNA (e.g., coyotes [ *Canis latrans* ] Eggert et al. 2003; African forest elephants [ *Loxodonta cyclotis* ] Kohn et al. 1999) but was extended to parents identified via genetic pedigree by Israel and May (2010) with application to green sturgeon ( *Acipenser medirostris* ).

Rawding et al. (2014) tested the method for Chinook salmon ( *Oncorhynchus tshawytscha* ) juveniles and compared it to a simple mark‐recapture estimate derived from combining adult carcass surveys and the same juvenile sample. This latter approach represents a form of the intercohort parent‐offspring close‐kin mark‐recapture as described by Bravington et al. (2016). Rarefaction estimates were tested using two different statistical approaches for estimating the curve's asymptote: the Beverton‐Holt model and a Continuous Smooth Hockey Stick as used by Kohn et al. (1999) and Eggert et al. (2003), respectively. Results suggested that the Beverton‐Holt model overestimated the number of spawners, consistent with the findings by Kohn et al. (1999) while the Continuous Smooth Hockey Stick approach worked well, provided sample sizes were large. In both cases, the findings concurred with those from previous simulation work (Petit and Valiere 2006). Drawing on the species richness literature, Sard et al. (2021) proposed and tested two additional non‐parametric approaches, the Chao (1984) and Jackknife (Quenouille 1949; Tukey 1958) estimators, under various population and sampling conditions using a purpose‐built simulation package. Their findings suggested that the Jackknife method was relatively accurate and precise when sample sizes were relatively high (e.g., when the number of offspring sampled is ≥ 90% of the number of total spawners), but consistently biased low with smaller relative sample sizes. By contrast, the Chao estimator was relatively unbiased across the range of sample sizes tested but exhibited high variability and correspondingly large confidence intervals when sample sizes were small relative to the true parental population. Ultimately, the authors concluded that the Chao method provided the most utility but cautioned end‐users to carefully assess sample sizes and species life‐history traits when interpreting results.

An emerging alternative to nonparametric estimators of species richness is the hierarchical Bayesian approach introduced by Royle et al. (2007), which uses data augmentation to estimate unobserved species (Kéry and Royle 2008). In this method, species survey results are modeled in a Bayesian framework where observation at a site is the product of both presence (i.e., the species exists to be sampled) and detection. The “augmentation” component refers to the addition of an arbitrary number of all‐zero detection histories to the input dataset, which essentially becomes a uniform prior for the true number of species present (Kéry and Royle 2008). Because presence is treated as a latent variable with a fixed length (the sum of observed and augmented detection histories), dimensionality is kept constant making Bayesian estimation feasible. Application to real‐world suggests that data augmentation can outperform the Jackknife estimator in both precision and repeatability (Kéry and Royle 2008). It has the added benefit of allowing co‐estimation of parameters for multiple sites, which enables the estimation of metacommunity dynamics (Dorazio et al. 2010). The approach has also been extended to capture‐recapture abundance estimation, where it has been similarly effective (Royle and Dorazio 2012).

Here we extend the work by Sard et al. (2021) to develop and test a hierarchical Bayesian pedigree rarefaction estimator employing data augmentation. This model uses a binomial likelihood incorporating separate “presence” and “observation” processes. To evaluate the new estimator's performance, we adapted the code developed by Sard et al. (2021) to provide a side‐by‐side comparison to both the previously tested Chao1 method and the improved iChao estimator (Chiu et al. 2014). We considered both accuracy and precision, as well as how both sex bias and errors in pedigree reconstruction affected the performance of all three approaches.

## 2 Materials and Methods

### 2.1 Simulations and Nonparametric Estimation

Pedigrees for this study were simulated using the R package *MATER* (https://github.com/nicksard/mater) in conjunction with code provided as a supplement to Sard et al. (2021) for which the package was developed. Functions within this package produce a breeding matrix of all potential mate pairs among a set of simulated females and males of total number *N*_P_, with the number of mates for each individual drawn from a Poisson distribution (λ = 4) and the number of offspring for each female drawn randomly from a uniform distribution of 2500–6500. These latter settings match those used in Sard et al. (2021) and were originally chosen to simulate typical salmonid reproductive ecology. The resulting set of simulated offspring can then be randomly sampled, with the breeding matrix used to determine the detected parents within each sample.

To directly compare our results with the previous work by Sard et al. (2021), we used their published code to simulate the same set of population and offspring sampling scenarios. This included assessing bias $\left({\hat{N}}_{P}/N_{P}\right)$, coefficient of variation (CV; $100\times \frac{\mathrm{SD}\left({\hat{N}}_{P}\right)}{\text{mean}\left({\hat{N}}_{P}\right)}$), and root mean squared error (RMSE; $\sqrt{\frac{1}{n}\sum_{i=1}^{n}\left({\hat{N}}_{P_{i}}-N_{P}\right)}$) across scenarios representing all potential combinations of a range of spawner numbers (*N*_P_ = 25, 50, 100, 200, 500) and offspring sample sizes (*N*_OBS_ = 25, 50, 100, 200, 500), with each population scenario simulated 1000 times. To assess the effects of both varying *N*_OBS_:*N*_P_ ratios and variation in the absolute scales of each variable, a further 1000 population and sampling simulations were run where *N*_OBS_ and *N*_P_ were each drawn from a uniform distribution of 25–500.

Finally, to evaluate the effect of pedigree reconstruction error we modified the simulated samples to create either false positives or false negatives (i.e., Type I or Type II error). We note that this differs slightly from the approach of Sard et al. (2021), who used the Type A and Type B errors proposed by Araki and Blouin (2005). This difference is because detection of shared or unique parents within a same‐cohort sample is essentially an exercise in sibship inference, and the parent‐based definitions of A and B (“fail to assign a true parent” and “assign an untrue parent”, respectively) therefore do not apply. Instead, we use the false positive and false negative terminology discussed in CKMR literature (e.g., Bravington et al. (2016)) which more generally applies to erroneously classifying or failing to classify a pair as “kin”. In this vein, we reasoned that the most common Type I error would be inferring a half‐sibling relationship between two individuals that did not share any parents (thereby reducing the number of detected parents by one) and that this was most likely when at least one individual had no other detected siblings. This is because the presence of other siblings would provide ancillary information that could increase the confidence of any inference, for example, if an individual has a detected full sibling they must share the same half siblings. Likewise, an individual cannot have more than two half siblings without at least one inter‐sibling sibship. We therefore subset the simulated sample to isolate individuals without any detected siblings (“true negatives”) and used random draws from a binomial distribution with probability equal to our desired Type I error rate to simulate error among the parents for those individuals (i.e., two draws per individual; one each for their simulated mother and father). Positive outcomes were randomly assigned to another parent, drawn from the overall pool of unique, correct‐sex parents within the sample. To simulate Type II error, we took much the same approach, isolating those individuals with at least one sibling match and using a binomial distribution to randomly assign “errors” to each of their parents. In this case, however, simulated errors consisted of reassignment to a new, unique parent not found elsewhere in the sample. We tested rates of 0%, 1%, and 5% for both types of errors using the same base settings for the initial comparison (*N*_P_ = 25, 50, 100, 200, 500; *N*_OBS_ = 25, 50, 100, 200, 500) and 100 replicates, with each error rate and type applied to the same set of simulated samples to ensure comparability.

For all simulated datasets, the Chao1 estimate of *N*_P_ was calculated using the package *SpadeR* (Chao et al. 2016). In brief, this method uses the total number of spawning adults detected in the sample set (*N*_DET_, see Table 1 for symbol definitions) in combination with the number of singletons (spawning adults detected as the parent of only one offspring; *a*_*1*_) and doubletons (spawning adults detected as the parent of exactly two offspring; *a*_*2*_) in the form:

(1) $${N_{P}}_{\text{Chao}1}=N_{\mathrm{DET}}+\frac{a_{1}\left(a_{1}-1\right)}{2\left(a_{2}+1\right)}\times \frac{\left(N_{\mathrm{OBS}}-1\right)}{N_{\mathrm{OBS}}}$$

**TABLE 1:** Mathematical symbols used in estimators compared by this study and their source (observed or estimated).

Variable	Definition	Estimator(s)	Source
*a1*	Singletons, that is, parents detect exactly once	Chao1, iChao	Observed
*a2*	Doubletons, that is, parents detect exactly twice	Chao1, iChao	Observed
*a3*	Tripletons, that is, parents detect exactly thrice	iChao	Observed
*a4*	Quadrupletons, that is, parents detect exactly four times	iChao	Observed
*L*	Total length of *y*, the data‐augmented detection vector and associate presence estimates *z*	Bayesian	Observed
*N*_*DET*_	Total number of parents detected in the sample	Chao1, iChao	Observed
*N*_*OBS*_	Total number of offspring sampled/observed	Bayesian, Chao1, iChao	Observed
*N*_*P*_	Estimated number of parents in the population	Bayesian, Chao1, iChao	Estimated
*p*	Probability of observing an adult present in the population	Bayesian	Estimated
*y*	Data‐augmented “detection” (≥ 1 for detected parents, 0 for augmenting additions)	Bayesian	Observed
*z*	Binary presence indicator for each data‐augmented detection history	Bayesian	Estimated
*ψ*	Probability of a supposed adult in the data‐augmented detection history being truly present	Bayesian	Estimated

With variance:

(2) $$\begin{array}{c} \mathrm{var}\left({N_{P}}_{\text{Chao}1}\right)=a_{2}\left[\frac{1}{4}{\left(\frac{N_{\mathrm{OBS}}-1}{N_{\mathrm{OBS}}}\right)}^{2}{\left(\frac{a_{1}}{a_{2}}\right)}^{4}+{\left(\frac{N_{\mathrm{OBS}}-1}{N_{\mathrm{OBS}}}\right)}^{2}{\left(\frac{a_{1}}{a_{2}}\right)}^{3}+\frac{1}{2}{\left(\frac{N_{\mathrm{OBS}}-1}{N_{\mathrm{OBS}}}\right)}^{2}{\left(\frac{a_{1}}{a_{2}}\right)}^{2}\right] \end{array}$$

This variance can then be used to calculate a 95% confidence interval using a log‐transformation to account for the general rightward skew of the Chao1 estimates:

(3) $$\begin{array}{c} \left[N_{\mathrm{DET}}+\frac{\left({N_{P}}_{\text{Chao}1}-N_{\mathrm{DET}}\right)}{e^{1.96\sqrt{\left(1+\frac{\mathrm{var}\left({N_{P}}_{\text{Chao}1}\right)}{{\left({N_{P}}_{\text{Chao}1}-N_{\mathrm{DET}}\right)}^{2}}\right)}}},\;N_{\mathrm{DET}}+\left({N_{P}}_{\text{Chao}1}-N_{\mathrm{DET}}\right)e^{1.96\sqrt{\left(1+\frac{\mathrm{var}\left({N_{P}}_{\text{Chao}1}\right)}{{\left({N_{P}}_{\text{Chao}1}-N_{\mathrm{DET}}\right)}^{2}}\right)}}\right] \end{array}$$

For the sake of completeness, we also tested the improved “iChao” formulation described by Chiu et al. (2014). This takes the previous estimate and adds information from tripletons (*a*_*3*_) and quadrupletons (*a*_*4*_), providing an estimate that is always greater than or equal to the Chao1 estimate:

(4) $$\begin{array}{c} {N_{P}}_{\text{iChao}}={N_{P}}_{\text{Chao}1}+\frac{a_{3}}{4a_{4}}\times \max\left(a_{1}-\frac{a_{2}a_{3}}{2a_{4}},0\right) \end{array}$$

With a 95% confidence interval constructed in the same fashion as the Chao1 estimator in (3). In both cases, however, we note that adjustments are necessary to deal with edge cases, for example, when *a*_*2*_ or *a*_*4*_ are equal to zero. The full derivations of each estimator are described in Chiu et al. (2014).

Sard et al. (2021) also compared the Jackknife estimator but concluded that the Chao1 estimator outperformed the Jackknife method in most scenarios and published a succinct comparison of the two. Given this conclusion, we modified the code to omit the calculation and comparison of Jackknife estimates.

### 2.2 Binomial Data Augmentation Model

To estimate *N*_P_, for each simulated cohort in a Bayesian framework, the number of pedigree‐derived “observations” (OBS) for each detected parent was first summarized into a vector *y* with total length equal to the number of detected parents. That is, a juvenile individual with no full‐ or half‐sibling matches to other juveniles from the same cohort would add two 1's to the detection vector, while a triad of half‐siblings would add a single 3 (for their shared parent) as well as three 1's. This vector was then “augmented” with an arbitrary number of 0's judged sufficiently large enough to be greater than the potential true value of N_P_ for a total length *L*. For this study, we set this to 50× the number of detected parents *N*_DET_, that is, $L=50N_{\mathrm{DET}}$. In practice, however, some consideration of a priori information about census size, which is presumably larger than *N*_P_, and experimentation will be required to set this appropriately, as values that are too small will unnecessarily bound the estimate of *N*_P_. At the same time, those that are far too large will be computationally inefficient and may hinder convergence.

Each observed and augmented detection *y*_*j*_ was modeled as the binomially distributed result of two processes: occurrence and detection. The first was represented by a latent state *z*_*j*_ representing the potential parent's existence and modeled by a Bernoulli trial with probability ψ:

(5) $$\begin{array}{c} z_{j}\sim \text{Bern}\left(\psi \right) \end{array}$$

While the second depended on the detection parameter *p*, with the overall number of detections modeled as the binomial result of both *ψ* and *p* with the number of draws equal to the number of juveniles captured from the cohort *N*_OBS_:

(6) $$\begin{array}{c} y_{j}\sim \mathrm{Bin}\left(z_{j}*p,N_{\mathrm{OBS}}\right) \end{array}$$

In the simplest case, *p* can be assumed to be constant among individuals as the offspring of all parents have an equal probability of being captured and, in turn, “detecting” their parents. However, this could be modified to incorporate covariates that affect both the fecundity of an adult and the probability of its offspring being captured. Both *ψ* and *p* were modeled as being Beta distributed with uninformative priors. Finally, the estimate of *N*_P_ is obtained by summing estimates for the occurrence process:

(7) $$\begin{array}{c} N_{P}=\sum_{j=1}^{L}z \end{array}$$

### 2.3 Computational Approach

We implemented the hierarchical Bayesian model in JAGS 4.3.0 (Plummer 2003) integrated with *R* using the *jagsUI* package (Kellner 2018). Each test was run using three Markov chain Monte Carlo (MCMC) chains run for an adaptation period of 1000 iterations, a burn‐in period of 2500 iterations, and a posterior distribution period of 5000 iterations with a thinning rate of 50. Parameter estimates were taken as the median of each posterior distribution rather than the mean to avoid issues with potential non‐normality. We evaluated chain convergence using the Gelman‐Rubin statistic *R̂* with successful convergence defined as *R̂* < 1.1 (Gelman and Rubin 1992). Credible intervals were calculated using the highest posterior density approach as implemented in the *HDInterval* (Meredith and Kruschke 2016). For efficiency, we parallelized both simulation and estimation using the *foreach* and *doParallel* packages (Weston and Calaway 2015).

## 3 Results

**FIGURE 1:**
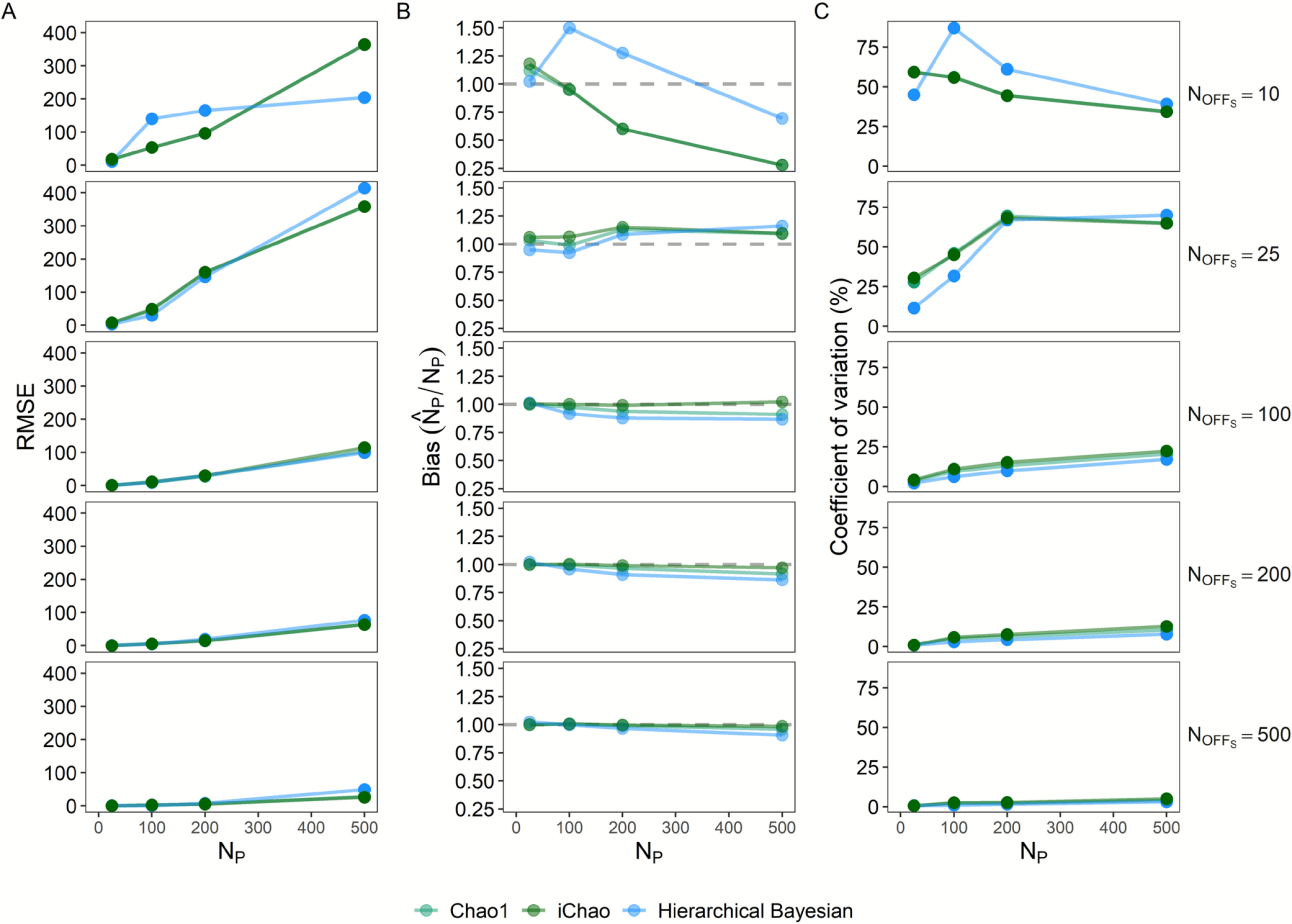
Performance of three pedigree‐based abundance estimators for all combinations of offspring sample sizes of 10, 25, 50, 100, 200, and 500 individuals and true adult abundances of 25, 50, 100, 200, and 500 individuals. Metrics include root mean squared error (RMSE) (A), bias (estimate/parameter) (B), and the coefficient of variation (C). The horizontal dashed line in panel B reflects the ideal unbiased estimate. Results represent 1000 simulation replicates.

Both the hierarchical Bayesian and iChao models performed well, providing estimates of *N*_P_ that were broadly in line with both those generated by the Chao1 estimator and the underlying true values. Notably, in simulations with a standard 1:1 sex ratio, both new estimators provided similar precision, with the Bayesian estimator displaying lower RMSE and CV values when the number of sampled offspring was low relative to the true number of parents (Figure 1A,C). The observed increase in precision was slight for the iChao estimator versus the Chao1 and marked for the Bayesian estimator. Precision metrics for all three methods converged as the number of sampled offspring rose, becoming relatively equal at an approximately 1:2 *N*_OBS_:*N*_P_ ratio.

**FIGURE 2:**
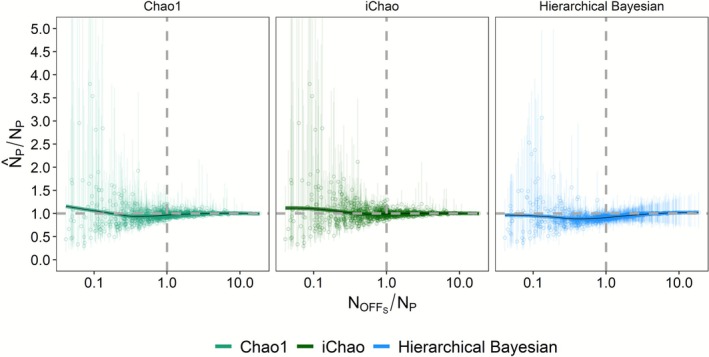
Realized bias (estimate/truth) for Chao1, iChao, and hierarchical Bayesian estimates of adult abundance relative to the ratio of sampled offspring versus adult abundance. Points reflect 1000 simulations where both the number of offspring sampled and the true adult abundance were drawn from a uniform distribution such that 25 ≤ *n* ≤ 500. Horizontal trends represent a loess smooth for each dataset and, in the case of the Bayesian estimator, are restricted to estimates passing the convergence threshold (R̂ ≤ 1.1). The horizontal dashed line represents the ideal unbiased estimate: Truth ratio while the vertical dashed line marks a 1:1 OBS:*N*_*P*_ ratio. Error bars depict 95% confidence intervals for the Chao1 and iChao estimates and 95% highest posterior density credible intervals for the Bayesian estimator.

Performance of the two general approaches differed most when the number of sampled offspring was especially low (e.g., in the range of 10 offspring samples). In this range, both Chao estimators tended to display a negative bias that deepened as the ratio of parental abundance to sampled offspring increased (Figures 1B and 2). By contrast, the hierarchical Bayesian estimator was positively biased at low adult abundances and negatively biased at high abundances, although to a lesser degree than the Chao estimators. At these sample sizes, the Bayesian estimator had greater RSME and CV than the Chao estimators indicating a consistent severe bias on the part of the latter with much greater variation on the part of the Bayesian estimator. On the other end of the scale, all methods were both relatively unbiased and highly precise when sample sizes were high relative to the adult abundance (1:1 or greater *N*_OBS_:*N*_P_).

The observed difference in patterns of bias and precision was also evident when estimates were made based on simulations where sample sizes and true adult abundances were drawn from a uniform distribution (Figure 2 and Figure S1). At low relative sampling depths, the average Chao estimates were biased high although this was likely due to the influence of a few outlier estimates. The iChao estimates displayed less bias than those calculated using the Chao1 approach and both methods were, on average, unbiased at *N*_OBS_:*N*_P_ ratios of 1:1 or greater, albeit with substantial variation among individual estimates. In turn, the hierarchical Bayesian estimator was approximately unbiased at low *N*_OBS_:*N*_P_ ratios, potentially due to less extreme positive outliers. However, at medium relative sampling densities (e.g., *N*_OBS_:*N*_P_ ratios of 0.5–5.0), Bayesian estimates were consistently biased low, on the order of 90% of the true value with relatively high precision versus the Chao estimates in that range. At all relative sampling depths, the Chao estimates were distributed on either side of the ideal unbiased line while the Bayesian method was much more likely to underestimate the true abundance than overestimate it. This skewed pattern accounts for the lower CV observed in the latter method and points to fundamental differences in how the two methods approach rarefaction estimation. Likewise, a marked difference was observed between the Chao 95% confidence intervals and the Bayesian 95% highest density posterior credible intervals. The former were much wider than the Bayesian credible intervals at low *N*_OBS_:*N*_P_ ratios but generally narrower at high ratios, where the Bayesian credible intervals approached a consistent pattern of 75%–150% of truth. This latter pattern belied the observed variation in Bayesian estimates, which was essentially unbiased at high *N*_OBS_:*N*_P_ ratios and likely stems from the remnant influence of uninformative priors on the posterior distributions. Interestingly, confidence/credible intervals for all three measures underperformed with the true values falling within the 95% confidence interval for 86.3% of Chao1 simulations and 67.4% of iChao simulations while the Bayesian 95% credible interval contained the true value for 83.4% of simulations.

**FIGURE 3:**
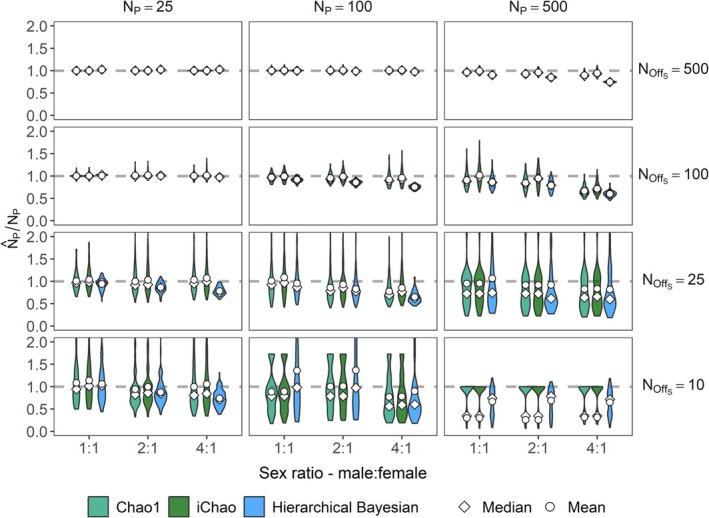
Violin plots of bias for the Chao and hierarchical Bayesian estimators applied to 100 simulations each of four offspring sample sizes, three true adult abundances, and three male: female sex ratios. The point within each violin reflects the mean value while the diamond reflects the median. The horizontal dashed line in each indicates the ideal unbiased estimate.

Performance of all three estimators diminished as sex ratios became increasingly skewed, but the effect was relatively small relative to the effect of relative sample size (Figure 3). In each case, the iChao estimator showed the least bias, albeit at the expense of greater variation in individual estimates. Mean estimates were consistently higher than median estimates for each category, highlighting the prevalence of outliers on the upper edge of the distribution for all methods.

**FIGURE 4:**
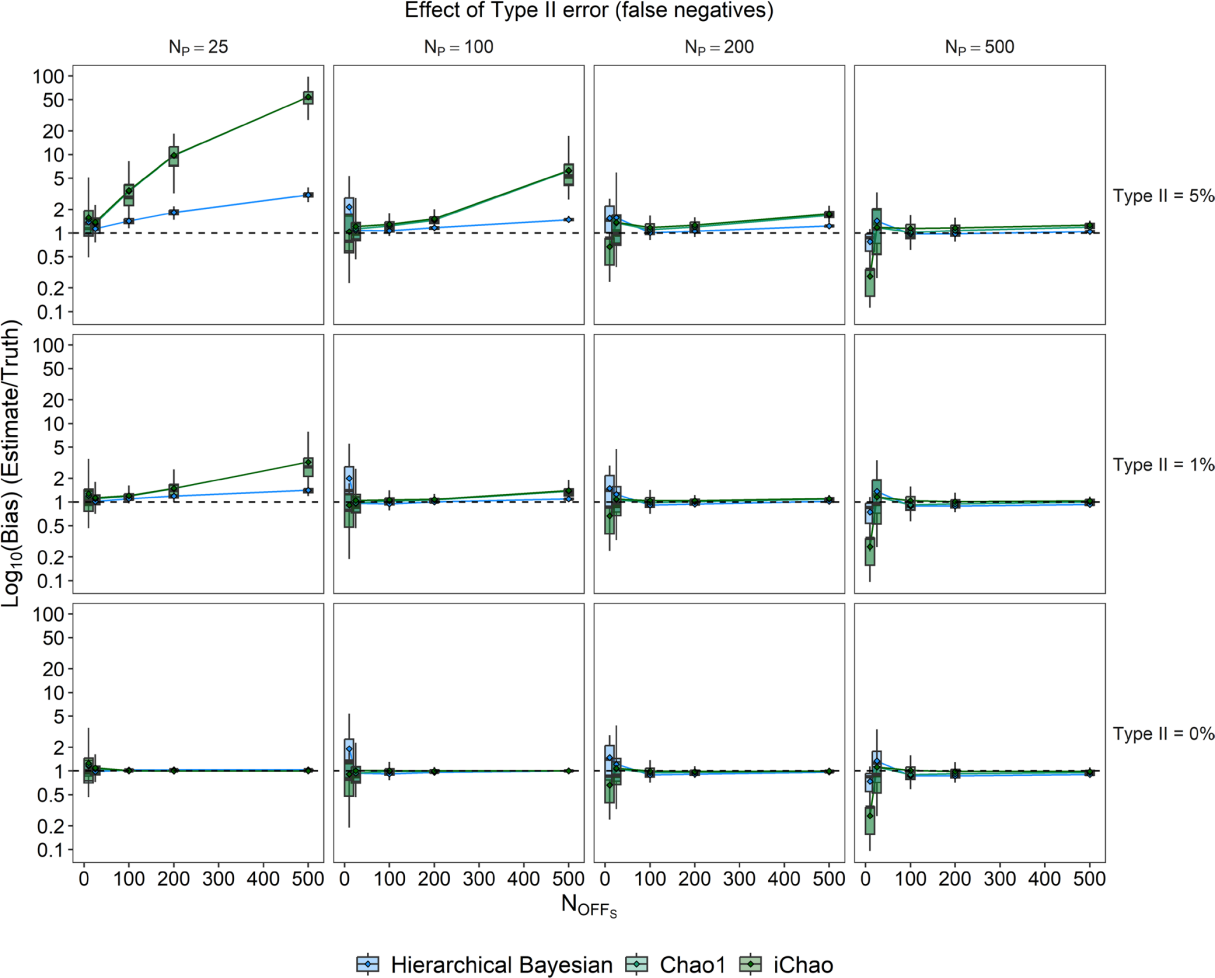
Effect of Type II errors (false negatives) on estimator bias (estimate/truth) for each of the three evaluated estimators and two rates of error: 0% (i.e., no error) and 5% for a range of sample sizes and true adult abundances. Boxplots depict the range of estimates while lines and points represent the mean values. All points and boxplots represent 100 simulation replicates with differing levels of error applied to the same samples. Note that the Y‐axis is on a log10 scale.

Conversely, the presence of simulated Type II error in pedigree reconstructions led to strong positive bias in both the Chao1 and iChao estimates, especially when sample sizes were large relative to the true adult abundance (Figure 4). The Bayesian estimator was much more robust to this effect; while bias was still observed, it was roughly half of that observed for the Chao estimators when the *N*_*OBS*_:*N*_*P*_ ratio was 4:1 (~1.5 vs. 3.0) and 1/25th (~2× vs. 50×) when the *N*_OBS_:*N*_P_ ratio was 20:1. Type I error was less influential, with all three estimators showing a very slight downward bias when the adult abundance was high and the *N*_OBS_:*N*_P_ ratio was low (Figure S2).

Convergence as assessed by R̂ was satisfactory for the majority of Bayesian estimates, although a handful (approximately 1%) of estimates at the extremes of the simulated *N*_OBS_:*N*_P_ ratios did not pass the threshold value of 1.1. Because there exists some controversy in the literature regarding the appropriateness of 1.1 as a cutoff, we also tested a threshold 1.05 and found that it excluded slightly more estimates with a corresponding reduction in bias and precision. This appeared to mainly be a consequence of the choice to use a fixed 10,000 iterations for the posterior distribution sampling in all *N*_OBS_:*N*_P_ ratio scenarios; when a subset of failed estimates were re‐run with a larger number of iterations (20,000), they converged satisfactorily albeit slowly (precluding our running all simulations with this higher iteration setting). The ability to assess convergence, either using R̂ or by visually inspecting trace plots, provides a supplementary measure of estimate confidence that is not available for the Chao estimators.

## 4 Discussion

Our results showed that approaches to pedigree rarefaction based on data augmentation produce relatively unbiased estimates and are potentially more precise under a wide range of conditions compared to the non‐parametric Chao estimators. This is not to say that the differences are stark; in our simulations, estimates for all methods converged on high accuracy as sampling depth increased, and mean estimates for the Chao1 and iChao methods were less biased in some edge cases, although with high variability in individual estimates. A notable exception of this was in the case of simulated pedigree reconstruction error, where our hierarchical Bayesian method was far more robust to simulated false negatives. This was most pronounced when sampling effort was high relative to the true adult abundance, exactly the scenario that simulations which assumed perfect pedigree reconstruction indicate should be the most accurate.

Coupled with the potentially higher precision of the hierarchical Bayesian method, this difference makes it a clear choice for real‐world applications where few estimates are likely to be made for any given population and pedigree reconstruction is likely to incorporate some level of error. The predictable patterns of bias we observed at low sampling depths additionally provide inbuilt indications of that bias that can be used to further caution or even potentially correct those estimates. We also observed an increase in accuracy and precision when comparing the iChao estimator to the previously tested Chao1 estimator, although this difference was slight relative to the differences noted between the Chao1 and hierarchical Bayesian estimators. Nevertheless, these results indicate that if a non‐parametric estimator is desired the iChao may perform better. However, our results also indicate caution when considering measures of uncertainty for all three estimators and especially the iChao; estimated 95% confidence/credible intervals contained the true value for only 83%–86% of simulations for the hierarchical Bayesian and Chao1 estimators and notably only 67% of the time for the iChao estimator. Thus, confidence/credible intervals for all three should be interpreted with some caution, especially when estimates indicate that the ratio of offspring samples is low relative to the parental abundance estimate.

The divergence in both robustness to pedigree reconstruction error and CV trends that we observed between the hierarchical Bayesian estimator and the non‐parametric Chao estimators is likely a reflection of fundamental differences in how the two methods operate. The Chao approach essentially estimates the limit of an asymptotic curve based on the shape of the ascending limb as inferred by the total number of species, as well as those observed exactly one or two times (Chao1) or one to four times (iChao). Thus, the resulting estimates are only constrained in that they cannot be less than the number of parents observed. By contrast, the hierarchical Bayesian approach models parental observations as the result of two processes, presence and observation probability, and uses the full range of observation values when doing so. This additional information and flexibility likely accounts for the greater precision as the full range of observation values is less likely to be skewed by random chance at low sampling depths than singletons and doubletons alone. However, the globally applied observation process and its uninformative prior are likely also responsible for a slight negative bias in estimates under all conditions. Given sparse sampling, the probability of observing any individual parent will be relatively low, approaching the lower limit of the beta distribution. This is quite different from the flat distribution provided by a uniform prior and, in practice, that prior will tend to “pull up” the parameter estimate without strong input data to the contrary. This, in turn, will negatively bias the presence estimate as the sampling results for a smaller population of more observable individuals can closely resemble that for a larger population of less observable ones without sufficient information to disentangle the two processes. Similarly, the observation process and usage of the full range of observation values also provide an opportunity for the model to incorporate the changes in “observability” that result from genotyping error. By contrast, the Chao estimators solely seek to estimate the asymptote of the accumulation curve and the presence of a non‐trivial false negative will present as a constant rate of additional parental accumulation at even the highest sampling intensities, resulting in the high positive biases that we observed.

In addition to performing well in terms of precision and accuracy, the parametric estimator we tested has the advantage of being based on a biologically relevant model structure with the observation component able to reflects individual‐level variation (e.g., fecundity) around a species‐ or population‐level mean. This provides an opportunity for information to be shared between systems, either as an informative prior or as shared parameters in a multi‐population model. Broadly, for a set number of parents, the probability that an individual parent will be “observed” in a single offspring random sample reflects that parent's relative contribution to the surviving individuals in the offspring's cohort at the time of sampling. This is ultimately the realization of a complex set of dependent processes that fall within the fields of reproductive success and recruitment. Because these vary considerably both among species and sexes due to life history differences and within species and sexes due to environmental conditions, they are unlikely to be able to be modeled explicitly. Nevertheless, because this probability partially reflects traits inherent to a species within a system, such as mean clutch size or correlated larval survival rates (such as might be observed for a nest‐spawning species), observation datasets from the same system across a range of years or different systems with similar conditions can likely inform each other. Likewise, because differences in observation probability within a species likely reflect the influence of the environment, these estimates may be of management utility in their own right. For example, wide posterior distributions for the observation parameter estimates would indicate a system with high variability in adult reproductive success. If this was observed to differ from other estimates for the same species (i.e., a population‐ rather than species‐level effect) it could indicate a system with especially patchy reproductive characteristics, for example, in salmonids this might indicate near‐total failure of many redds, in turn suggesting habitat limitations or egg predation associated with wholesale redd loss rather than the more evenly distributed larval mortality that would occur later in life. A parallel exists in the species richness field, where community models can encompass individual patches with presence and observation varying among species, with the former also a function of habitat and the latter a function of survey (Zipkin et al. 2010). This potential for information‐sharing and the biologically meaningful structure are core advantages of the hierarchical approach.

More broadly, estimating *N*_P_ can be influenced by a range of reproductive ecology factors, including fecundity distributions and associated quality metrics, fertilization rate, mating system and the adult operational sex ratio. Notably, the connection between sampled offspring from a cohort and the estimable *N*_P_ becomes increasingly biased low as the ratio of *N*_P_ and their viable offspring approaches zero, and as the time since fertilization increases. In effect, this causes families to “drop out” from the pool of surviving offspring in a cohort, rendering them unobservable. Finally, spatial and temporal variation in offspring production relative to sampling can influence estimates, especially in systems with some level of offspring dispersal. Accurate implementation of pedigree‐based estimation therefore requires careful consideration of a species' and population's particular life history characteristics and how they may interact with both reproduction and sampling. The potential for such interactions represents a possible pitfall in the application of pedigree‐based estimators but is also an opportunity in that inferred pedigrees contain information regarding ecological dynamics such as mating behavior and dispersal that are often hard to observe using other approaches. Development of more complex models that take advantage of multiple sampling years or locations may therefore be able to elucidate a wealth of additional biological and ecological details.

Ultimately, our results demonstrate that the hierarchical Bayesian approach to pedigree accumulation using data augmentation represents an accurate, precise, and robust estimator of spawning stock abundance, that is, the number of parents for a cohort, using within‐cohort samples in a field thus far dominated by methods that either require cross‐cohort sample sets or produce estimates of effective rather than census size (Luikart et al. 2010; Waples and Feutry 2022). As highlighted by Rawding et al. (2014), Sard et al. (2021), and others, this broadens the applicability of kinship‐based approaches to semelparous species where sampling parent‐offspring pairs may be infeasible and cross‐cohort siblings are nonexistent. Beyond this relatively small (but economically and socially important) set of species, however, the introduction of a parametric within‐cohort estimator may present an opportunity to develop models that join within‐ and cross‐cohort observations. Such a joint model could reduce uncertainty by more efficiently incorporating the range of available samples and potentially extend the range of actionable model outputs by incorporating observation‐based estimates of relative recruitment success. Validation of a parametric pedigree accumulation estimator, therefore, extends an increasingly powerful set of kinship‐based abundance estimation tools and provides a foundation for future innovation.

## Author Contributions

**Benjamin Marcy‐Quay:** conceptualization (lead), formal analysis (lead), methodology (lead), software (equal), visualization (lead), writing – original draft (lead), writing – review and editing (equal). **Nicholas M. Sard:** methodology (supporting), software (equal), writing – review and editing (equal).

## Conflicts of Interest

The authors declare no conflicts of interest.

## Supporting information


**Appendix S1:** Supporting information.

## Data Availability

All data used in this study were simulated using the code available for review at: https://doi.org/10.5061/dryad.02v6wwqhf.
