# Supplementary material for: Estimation of Parental Abundance Using Hierarchical Bayesian Modeling With Data Augmentation
Source: Ecol Evol. 2026 Mar 12;16(3):e73131. doi: 10.1002/ece3.73131 (PMC13093385; doi:10.1002/ece3.73131)
Supplement: Supplementary file 1 — Appendix S1: Supporting information. [file ECE3-16-e73131-s001.docx]

**Supplementary material**


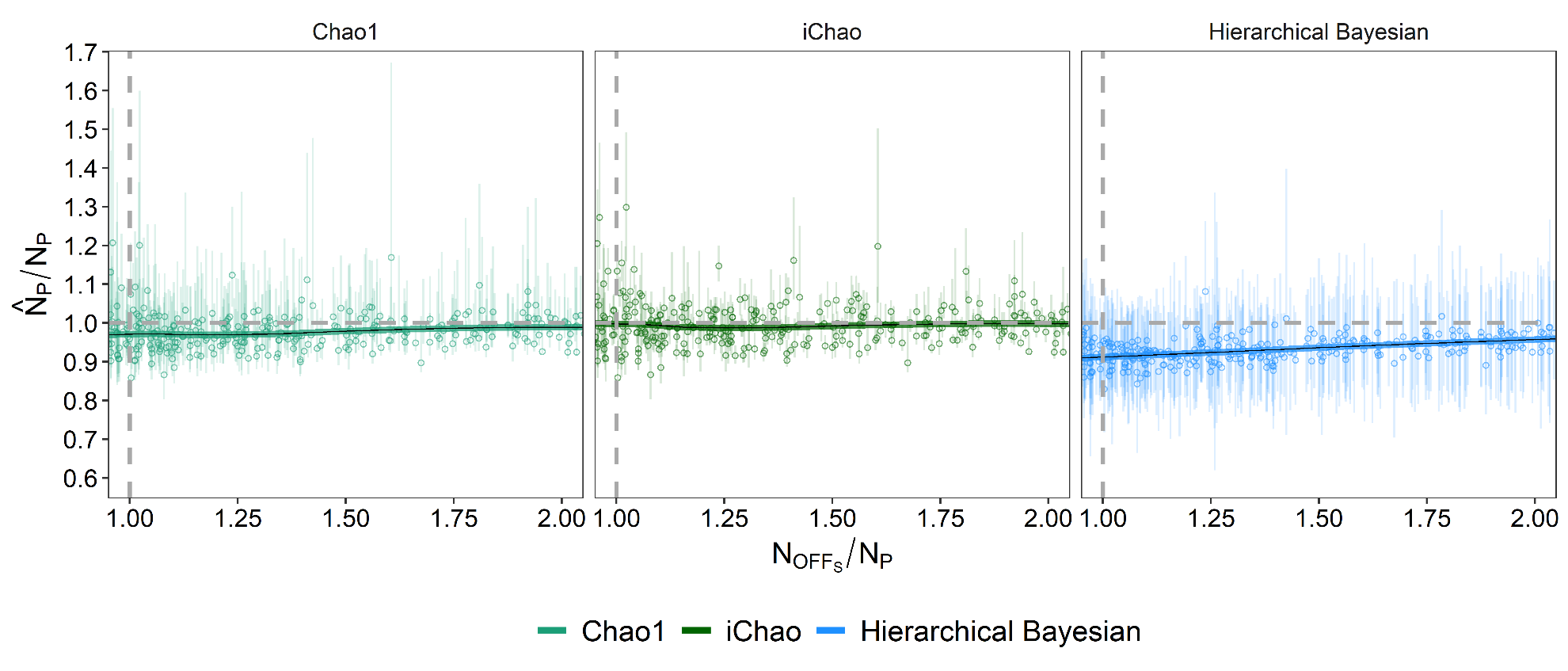


Figure S1. Enlarged view of the realized bias (estimate/truth) for Chao1, iChao, and hierarchical Bayesian estimates of adult abundance for ratios of sampled offspring to adult abundance of 1-2. Horizontal trend represent a loess smooth for each dataset and, in the case of the Bayesian estimator, are restricted to estimates passing the convergence threshold (R̂ ≤ 1.1). The horizontal dashed line represents the ideal unbiased estimate:truth ratio while the vertical dashed line marks a 1:1 OBS:*N_P_* ratio. Error bars depict 95% confidence intervals for the Chao1 and iChao estimates and 95% highest posterior density credible intervals for the Bayesian estimator


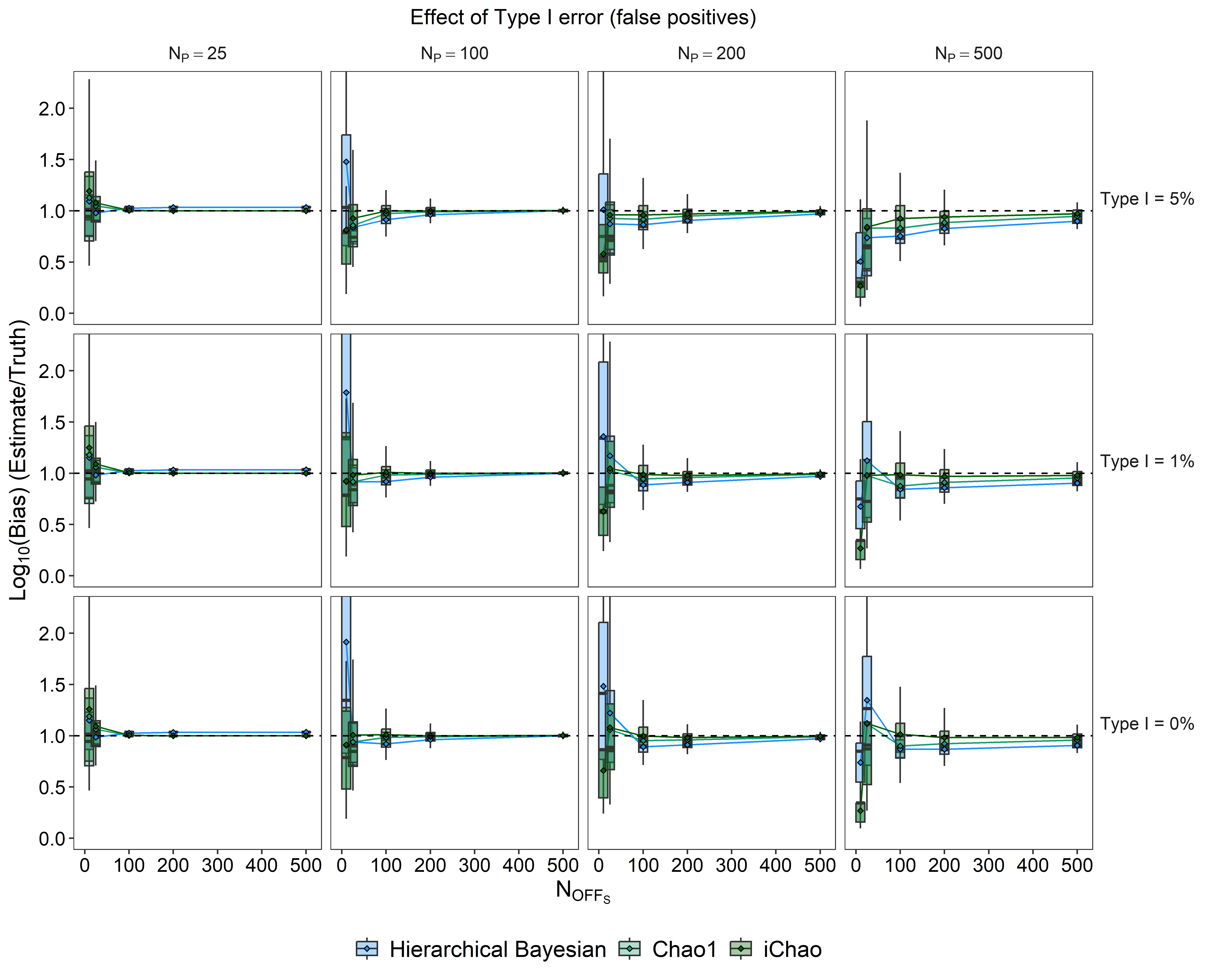


Figure S2. Effect of Type I errors (false positives) on estimator bias (estimate/truth) for each of the three evaluated estimators and three rates of error: 0% (i.e., no error), 1%, and 5% for a range of sample sizes and true adult abundances. Boxplots depict the range of estimates while lines and points represent the mean values. All points and boxplots represent 100 simulation replicates with differing levels of error applied to the same samples.
